# Dynamic Instability and Time Domain Response of a Model Halide Perovskite Memristor for Artificial Neurons

**DOI:** 10.1021/acs.jpclett.2c00790

**Published:** 2022-04-22

**Authors:** Juan Bisquert, Antonio Guerrero

**Affiliations:** Institute of Advanced Materials (INAM), Universitat Jaume I, 12006 Castelló, Spain

## Abstract

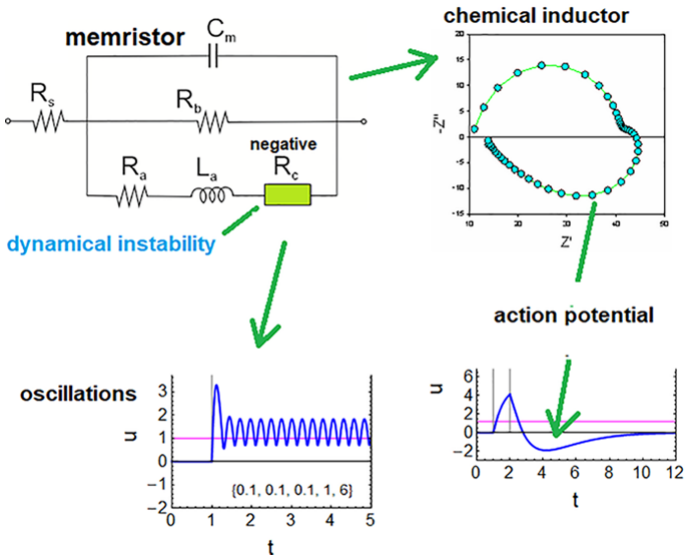

Memristors are candidate devices for constructing artificial neurons, synapses, and computational networks for brainlike information processing and sensory-motor autonomous systems. However, the dynamics of natural neurons and synapses are challenging and cannot be well reproduced with standard electronic components. Halide perovskite memristors operate by mixed ionic–electronic properties that may lead to replicate the live computation elements. Here we explore the dynamical behavior of a halide perovskite memristor model to evaluate the response to a step perturbation and the self-sustained oscillations that produce analog neuron spiking. As the system contains a capacitor and a voltage-dependent chemical inductor, it can mimic an action potential in response to a square current pulse. Furthermore, we discover a property that cannot occur in the standard two-dimensional model systems: a three-dimensional model shows a dynamical instability that produces a spiking regime without the need for an intrinsic negative resistance. These results open a new pathway to create spiking neurons without the support of electronic circuits.

Information processing and sensory data management in networks of neurons and synapses in the brain occur by the stimulation of neurons causing repeated action potentials in periodic rhythms.^1−3^ To construct artificial brainlike computation and sensory-motor autonomous systems, we need networks of miniature elements that perform and distribute rhythmic spiking.^4−7^ Currently, many CMOS-based neuromorphic computation systems use very simple integrate-and-fire neurons.^8^ These consist basically of an RC (resistance capacitor) circuit that becomes progressively charged and discharges suddenly when the voltage exceeds a threshold value.

However, natural spikes have more complex properties such as a refractory postspike period.^9^ Natural neuron spiking is a self-sustained oscillation connected to dynamical instability.^3^ A Hopf bifurcation is a critical point where the system destabilizes and a periodic behavior that never reaches equilibrium arises.^10,11^ The spiking patterns are well described by dynamical models formed by a few differential equations based on the pioneering work of Hodgkin and Huxley on the giant axon of the squid.^2^ Many useful simplified models with a smaller number of equations, such as the FitzHugh–Nagumo model, have been derived^11−15^ and classified by the number and type of equations and the dynamic and bifurcation properties.^11^ We have recently described the conditions for Hopf bifurcation and spiking regimes in two-dimensional models using the methods of equivalent circuits (EC) and impedance spectroscopy.^16^ We concluded that the main elements needed for a self-sustained oscillation to occur are (1) a membrane capacitor, (2) a chemical inductor, and (3) a built-in negative resistance.^17^

A memristor is a two-terminal device whose resistance depends on the history of current and voltage applied to the device. Memristors allow the storage of information by the metastable modification of device conductivity.^18−22^ Memristor devices are the main candidates for producing compact and reliable artificial neurons and synapses for computational algorithms based on neuron spiking.^9,18,19,22−27^ Recently, halide perovskite memristors have been investigated because their ionic–electronic properties and strong hysteresis effects are promising for neuromorphic applications.^18,19,28−30^ Halide perovskites produce synapselike functionality with a simple structure and extremely low energy consumption.^26,31^ The question is, can we generate the analog neuron properties of spiking neurons with memristors? This property requires the presence of instabilities in addition to the memory conductance effects.

To address this topic, we use here a model for a halide perovskite memristor that has recently been shown to describe the experimental current–voltage cycling and impedance response well.^32^ This type of model may be applied with suitable adaptations to a variety of material platforms such as mixed ionic–electronic organic materials.^5,9,33^ Here we analyze the time transient voltage response to step stimulation, and we derive the stability and the bifurcation properties of this model using the EC methods.^16,17^

The model will be presented in different steps. We first describe a two-dimensional simplified version of the model that enables the calculation of the transient response to a voltage or current pulse as is usually done for the analysis of synapsis potentiation and plasticity.^26,30,34,35^ Then we present the three-dimensional model and show that it presents a dynamical instability in which self-sustained oscillations occur without the need for an intrinsic negative resistance, in contrast to the two-dimensional models.

*Two-Dimensional Model*. We discuss the dynamical memristor model formed by the system of equations^32^
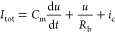
1
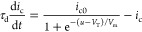
2

The model has three independent variables: *I*_tot_ and *u* are the external current and voltage, and *i*_c_ is an internal current. Equation 1 describes the three components of *I*_tot_: a capacitive charging of the interfaces with capacitance *C*_m_, a small ohmic current of constant resistance *R*_b_, and the slow internal current *i*_c_ described by eq 2. As described before,^32^eq 2 represents a diffusion or migration time of ions that introduces a delay of *i*_c_ with respect to the external perturbation by the characteristic time constant τ_d_. In the steady state, the slow current takes the value
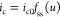
3according to the function
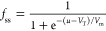
4that varies from 0 at low voltage to 1 at high voltage, with the redox potential *V*_T_ and an ideality factor *V*_m_ with dimension of voltage. Consequently the slow current varies from 0 to a saturation value *i*_*c*_0__ depending on the applied voltage. Specific physical mechanisms behind the function *f*_ss_ are the filamentary conductive pathway^20^ or the decrease in a surface barrier between the perovskite layer and the contacts.^36,37^ However, filamentary systems usually show an abrupt transition to the high conduction state, and the above model is adapted to those systems that show a gradual transition.

Combining eqs 1 and 3, we find that the steady state current–voltage equation is
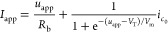
5The total current of the memristor (gray line) and the component currents are shown in Figure 1a.

**Figure 1:**
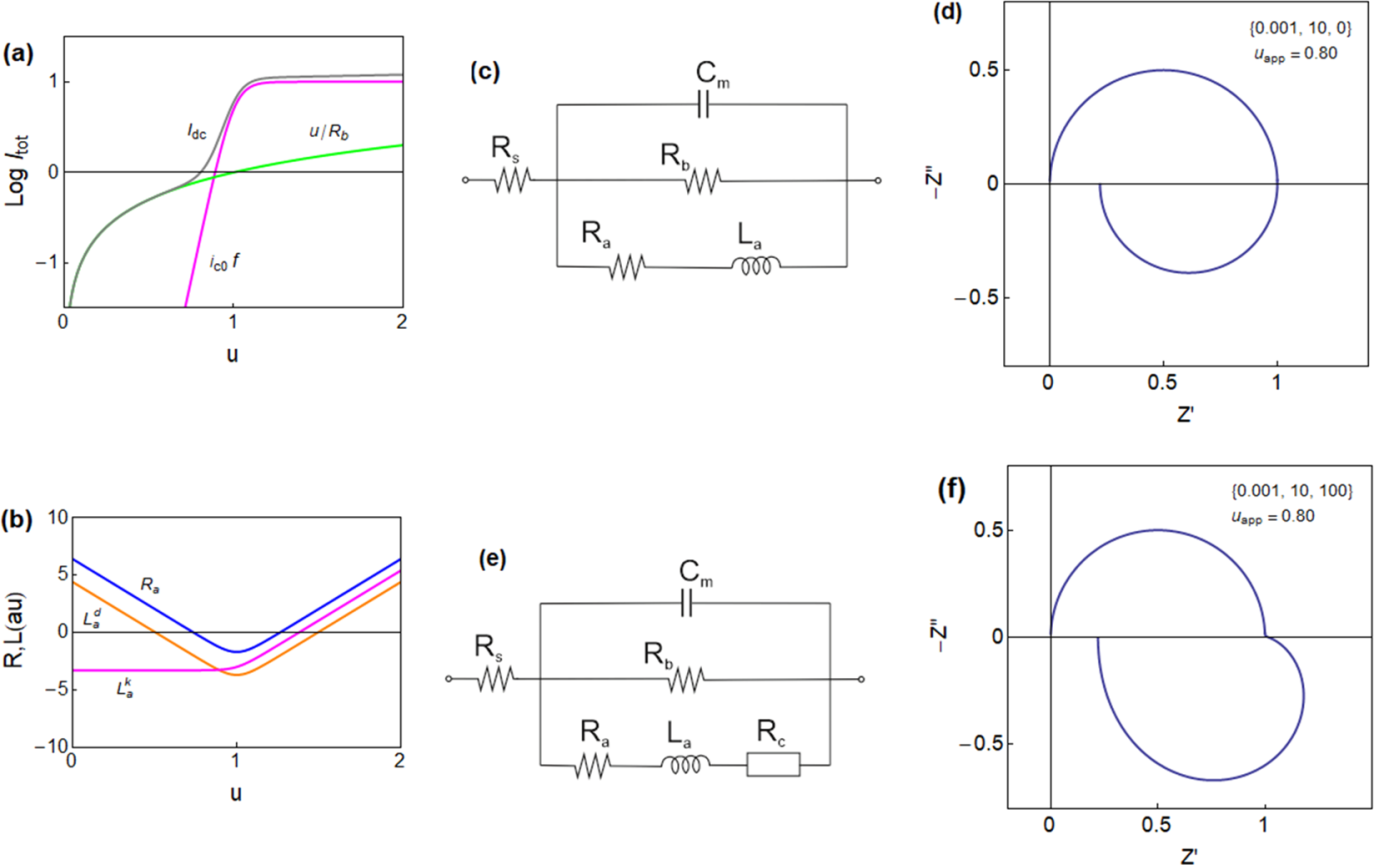
(a) Logarithmic current–voltage curve for the model memristor, showing the two component currents and the total equilibrium dc current (gray line). (b) Impedance parameters. (c) Equivalent circuit of eqs 1 and 2 and (d) the impedance spectrum under the indicated conditions. (e) Equivalent circuit of eqs 1, 10, and 11 and (f) the impedance spectrum. Parameters: *R*_b_ = 1; *i*_*c*_0__ = 10, *V*_T_ = 1, *V*_m_ = 0.05, and [τ_m_, τ_d_, τ_k_].

By the linearization of eqs 1−2 at a steady-state point, we can obtain the impedance spectroscopy response function in terms of the variable *s* = iω, where ω is the angular frequency of the small perturbation. The impedance model is
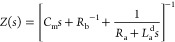
6
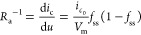
7
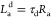
8The model of eq 6 is the recently described impedance of a chemical inductor.^17^ It is characteristically observed in halide perovskite devices in the high-voltage domain.^38,39^ The equivalent circuit is shown in Figure 1c, impedance parameters are shown in Figure 1b, and the characteristic spectrum with the inductive component in the fourth quadrant is shown in Figure 1d. The interpretation of EC elements of Figure 1c is as follows. *C*_m_ is a capacitance element as already mentioned. In the halide perovskites, there are two dominant capacitances.^32,39,40^ The geometric capacitance *C*_g_ stands for dielectric relaxation at high frequency, and it is independent of the voltage. On the other hand, a low-frequency capacitance *C*_1_ is related to the ionic polarization of the interface. *C*_1_ is voltage- and light-dependent and takes very large values. The resistance and inductor elements, *R*_a_ and *L*_*a*_^*d*^, respectively, are the components of the chemical inductor branch in the equivalent circuit. These elements are formed by the delay equation,^17^ in our case eq 2, that is interpreted as an electronic current that depends on ionic displacement.^32,41^

*Time Transient Response of the Two-Dimensional Model*. We analyze the time transient response of the model to a square perturbation, which was not studied in the previous publication.^32^ This analysis is particularly important for the formation of the analog response of artificial neurons and synapses. In the experiments, the sample is pulsed repetitively and the changing response is recorded.^30,34^ The output in response to a pulsed perturbation can be obtained by the solution of eqs 1 and 2. The transient response is controlled by two main time constants in the model: τ_m_ = *R*_b_*C*_m_ and τ_d_, and by the pulse duration time Δ*t* so that different responses to a step perturbation are expected, according to their combinations. A representative set of responses to a square current perturbation *I*_app_ with *R*_s_ = 0 is shown in Figure 2. The different behaviors can be interpreted in terms of the model EC of the chemical inductor of Figure 1c. Note, however, that the circuit is strictly valid only for a small perturbation. When the large external perturbation is applied, the circuit elements are not constant but undergo the variations shown in Figure 1b. In a detailed analysis, the response times include the total resistances of the network instead of the simplified time constants τ_m_ and τ_d_. The calculation tool used to explore all of the possibilities is provided in the Supporting Information.

**Figure 2:**
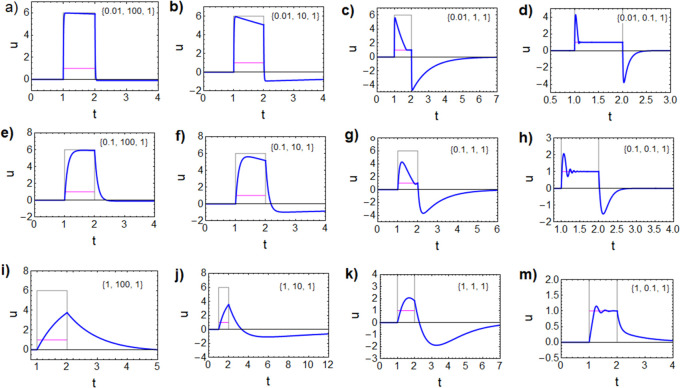
Time transient response to the current step value *I*_app_(*u*_app_) and duration Δ*t*. The gray line is the current pulse indicated by the voltage *R*_b_*I*_app_. The magenta line is the pulse voltage at equilibrium *u*_app_ = 1. [τ_m_, τ_d_, Δ*t*] is as indicated, and *R*_b_ = 1; *i*_*c*_0__ = 10 in all cases.

In Figure 2, the capacitor *C*_m_ is charged within the time constant τ_m_, producing a voltage *I*_app_*R*_b_ (indicated by the gray line). The inductor line responds with the time constant τ_d_ = *L*_a_^d^/*R*_a_. The magenta line is the final equilibrium voltage of the activated state with the dc resistance
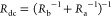
9In Figure 2a, we start with a very short τ_m_ charging time. The current rises instantaneously to the gray level, and because the inductor is large, the magenta line will be achieved only at extremely long times. For smaller inductor values in Figure 2c,d, we observe the inductive negative spike reaching the dc voltage value of the square pulse and a negative discharge characteristics when the pulse is switched off. In Figure 2e, we combine a longer charging time and a very large inductor. The response is the typical RC charging process to the gray reference and subsequent discharge. For smaller inductor values in Figure 2f,g, the decay of the initial current peak reaches the stationary line. For the very small inductor value in Figure 2h, the system displays overdamped oscillations.

By using a larger τ_m_ (or a shorter pulse time), in Figure 2i the signal is not allowed to reach the gray saturation value. Then for smaller inductor values shown in Figure 2f, we have a rising feature in charge but a negative spike in discharge due to the chemical inductor. This pattern closely reproduces the natural shape of the action potential in biological neurons. This action potential is associated with a single square perturbation and not self-sustained oscillations; we note that the oscillations are damped and vanish in Figure 2h–m. The general conditions for obtaining stationary oscillations in a two-dimensional dynamical model have been reviewed,^16^ and it has been concluded that an internal negative resistance sector under the dc condition is necessary to destabilize the system and produce a Hopf bifurcation, as in the FitzHugh–Nagumo model.^12^ Instabilities and bifurcation can happen in two-dimensional models in the presence of time-delayed coupling,^42^ which is not considered here. Consequently, the model of eqs 1 and 2 cannot produce a bifurcation and limit cycle oscillations because it lacks the negative resistance feature.

For the calculation of the transient current to a step of the external voltage *V*_app_, we add the voltage drop across the series resistance according to
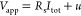
10The results are shown in Figure 3.

**Figure 3:**
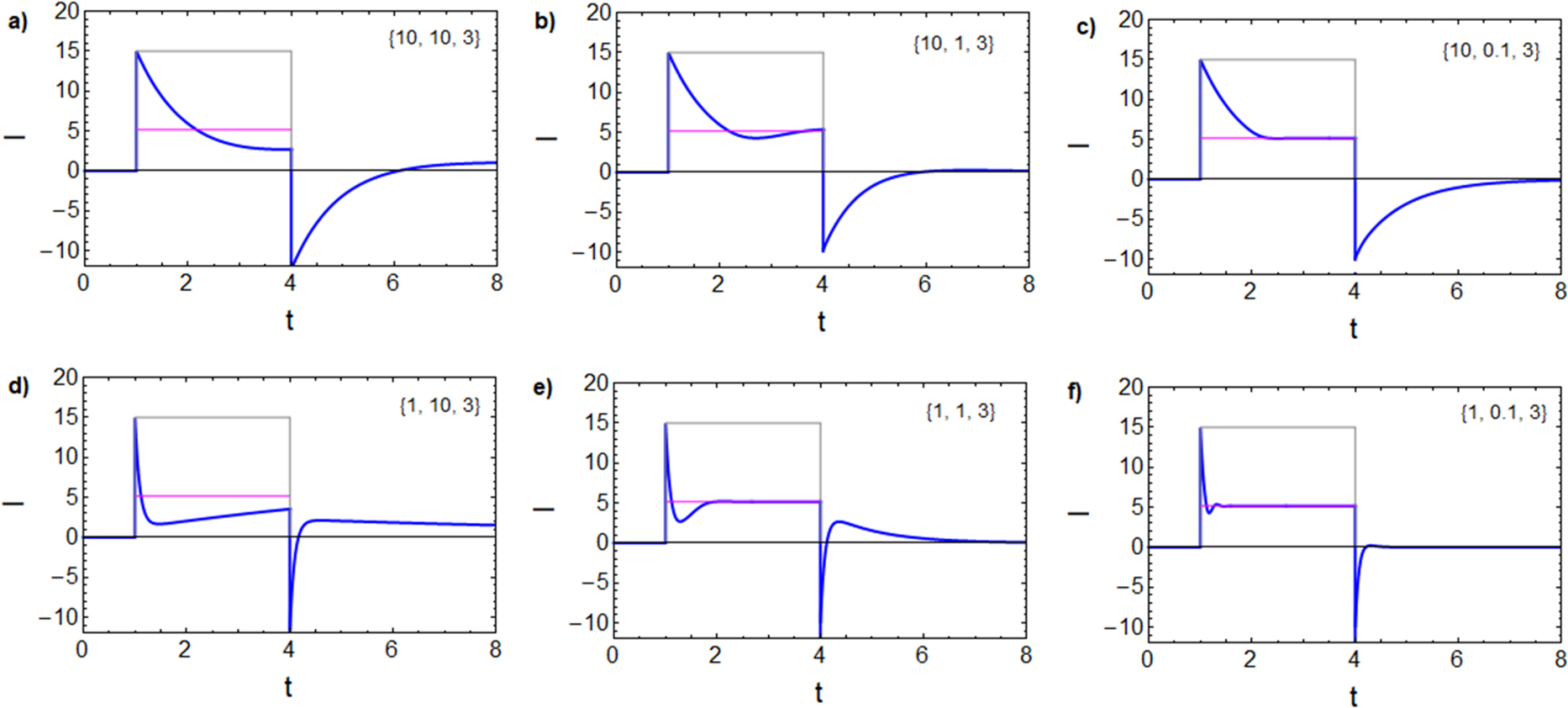
Time transient response to the voltage step value *V*_app_ = 1.5 V and duration Δ*t*. The gray line is the initial value of the current when the applied voltage lies at the series resistance, *V*_app_/*R*_s_, and the magenta line is the pulse current at equilibrium, *I*_app_ = *I*_tot_(*V*_app_). [τ_m_, τ_d_, Δ*t*] as indicated, *R*_s_ = 0.1, *R*_b_ = 1, and *i*_*c*_0__ = 10 in all cases.

*Three-Dimensional Model*. We proceed to a more general model as described previously.^32^ In this model, we again use eq 1 but introduce two slow variables, *i*_c_ and *f*, described by the following equations:
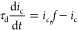
11
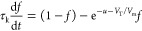
12

Equation 12 describes the voltage-controlled activation of the high conduction configuration. τ_k_ is the characteristic formation time of the high conduction state. The variable *f* is an occupation function (0 ≤ *f* ≤ 1) that describes the onset of the memristor activated state. Similar to the ion channel behavior in neurons,^2^ the variable *f* indicates the state of the mechanism that establishes the high conductivity state in the memristor. As commented on for eq 2, the parameter τ_d_ in eq 11 indicates the diffusion time necessary to establish the configuration of high *f* that produces the large electronic current *i*_*c*_0__.

If we consider the steady-state situation, we again obtain eqs 3 and 4, and the current voltage is given by eq 5. If we assume that the formation time τ_k_ is short, then eq 12 leads to the quasi-equilibrium condition *f* ≈ *f*_ss_ and the model becomes the two-dimensional set of eqs 1 and 2 that we analyzed before. In the three-dimensional model, the transient response is controlled by three main time constants (τ_m_ = *R*_b_*C*_m_, τ_d_, and τ_k_) and by the pulse time duration.

The impedance of the three-dimensional model has the form^32^
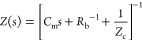
13

The *Z*_c_ impedance is
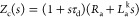
14

These last two equations provide the EC of Figure 1e. The impedance parameters are defined as before, and a new inductor appears because of eq 12
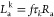
15

We can write eq 15 as
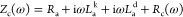
16where
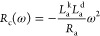
17

The impedance spectra have been fully described,^32^ and a representative example showing an RC arc at high frequency and a distorted inductive arc at low frequency is shown in Figure 1f. This type of spectrum has been observed experimentally for halide perovskite memristors,^32^ giving strong support to the relevance of the model.

*Bifurcation and Self-Sustained Oscillations*. In Figure 4, we show the impedance spectra of the general three-dimensional model (eqs 1, 11, and 12) for a different set of kinetic parameters in comparison to Figure 1f. Figure 4a is the normal form of the inductive spectrum of a chemical inductor shown in Figure 1d,^17^ but Figure 4b presents a negative resistance at a finite frequency. This pattern occurs in a narrow voltage range around the transition region of the memristor, as discussed later. The spectrum of Figure 4b is the characteristic mark of a Hopf bifurcation that causes self-sustained oscillations in electrochemical systems and neurons under galvanostatic conditions.^12,16,43^

**Figure 4:**
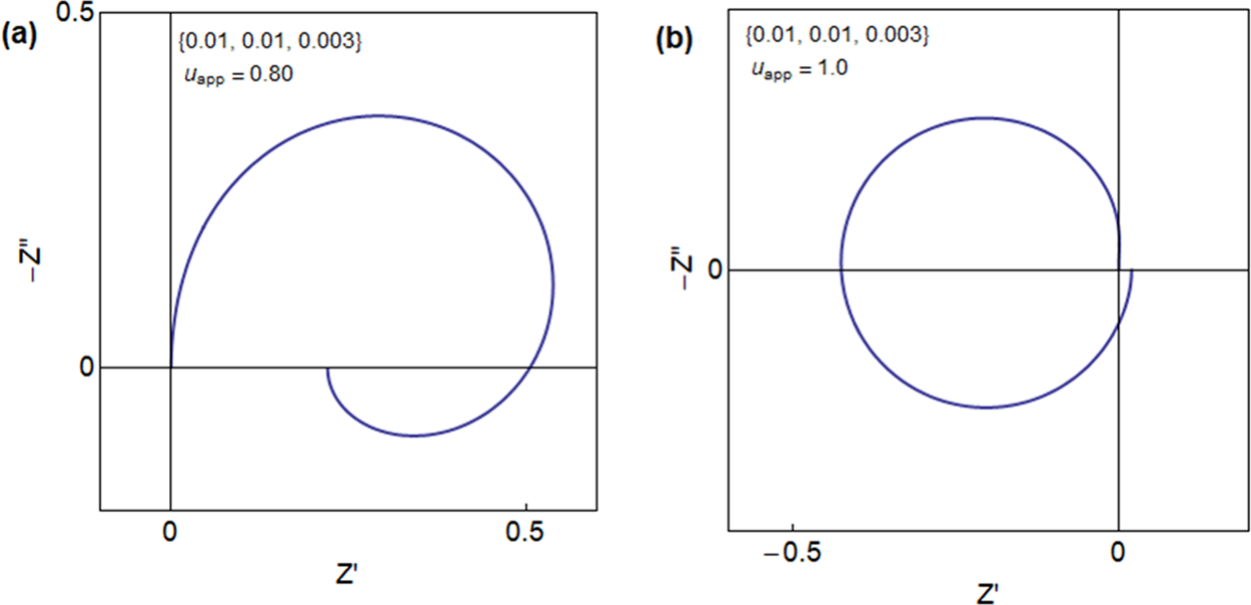
Impedance spectra of the model memristor of eqs 1, 10, and 11 under the indicated conditions. Parameters: *R*_b_ = 1, *i*_*c*_0__ = 10, *V*_T_ = 1, *V*_m_ = 0.05, and [τ_m_, τ_d_, τ_k_].

Our model, however, does not contain a negative resistance component, as already mentioned, because the *I*–*u* curve in Figure 1a is formed by strictly positive dc resistances. In the present system, the negative resistance is caused by the coupling of two inductive features that produce the element *R*_c_(ω) in eq 17. It is a dynamical instability that exists at nonzero frequency and disappears under dc conditions.

Because the reported general analysis of bifurcation by IS^16^ is restricted to two-dimensional systems, we apply the normal mode method^10^ to the current system. From linearized eqs 1, 11, and 12, we find the Jacobian
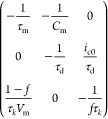
18

The characteristic equation for the eigenvalues λ has the form
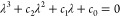
19where the coefficients are given by the expressions
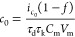
20
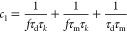
21
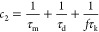
22

At the Hopf bifurcation, a pair of eigenvalues become purely imaginary.^44^ We insert the form λ = iϖ and obtain the equations
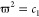
23
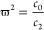
24

Therefore, the Hopf bifurcations occur at the points that satisfy
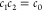
25

The different coefficients as functions of voltage are plotted in Figure 5a. It is observed that in the crossing of *c*_0_ and *c*_1_*c*_2_ two Hopf bifurcations occur that mark a domain where the system is unstable and can perform limit cycle oscillations. By solving the full three-dimensional system (eqs 1, 11, and 12) in the time domain, the oscillations can be generated as shown in Figure 5b,c.

**Figure 5:**
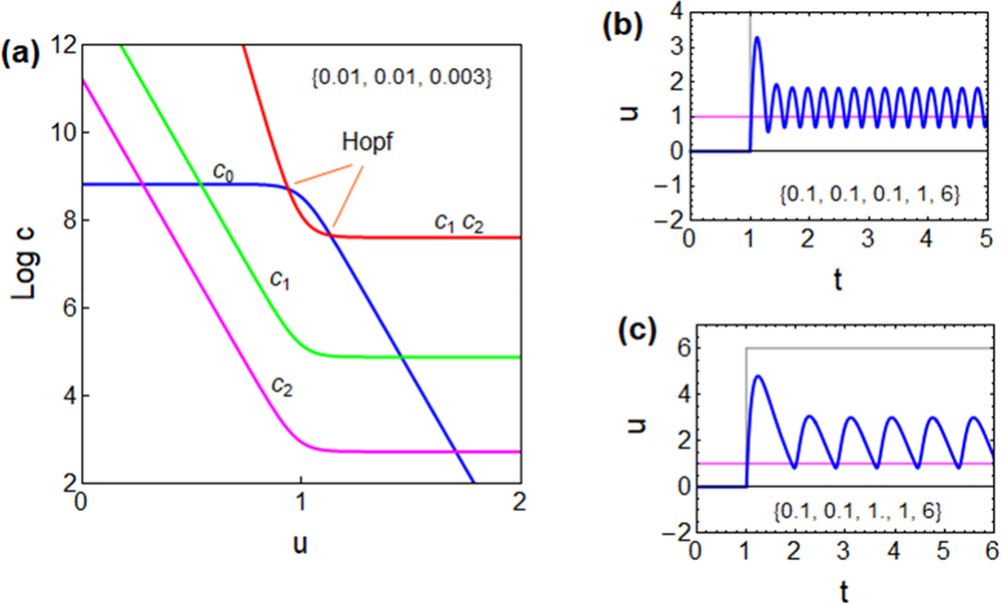
Model of eqs 1, 10, and 11. (a) Coefficients of the characteristic equation vs voltage and location of the Hopf bifurcations. [τ_m_, τ_d_, τ_k_] is as indicated. (b, c) Time transient response to current steps of value *I*_app_(*u*_app_). The gray line is the current pulse indicated by the voltage *R*_b_*I*_app_. The magenta line is the voltage at equilibrium *u*_app_. [τ_m_, τ_d_, τ_k_, *u*_app_, *I*_app_] is as indicated. *R*_b_ = 1; *i*_*c*_0__ = 10 for all cases.

In conclusion, we analyzed the time domain and impedance response of a three-dimensional memristor dynamical model. In a simplified two-dimensional version, we obtained responses to a pulsed current perturbation in which the main properties of a neuronal action potential can be reproduced with a simple device without internal electronic parts.

In addition, we analyzed the properties of oscillating dynamics. It has been previously established that two-dimensional models require an intrinsic negative resistance, which persists under the dc condition, to produce a Hopf bifurcation that moves the system into self-sustained oscillations. Here we find a new property: in the three-dimensional system, a Hopf bifurcation occurs without an intrinsic negative resistance. This is because the product of inductors produces a dynamical negative resistance that exists only under transient conditions. So far, such oscillating patterns of the perovskite memristor have not been observed.

## Supporting Information Available

Mathematica program for the calculation of transient responses and impedances (PDF)
