# Supplementary material for: Dynamic Instability and Time Domain Response of a Model Halide Perovskite Memristor for Artificial Neurons
Source: J Phys Chem Lett. 2022 Apr 22;13(17):3789–95. doi: 10.1021/acs.jpclett.2c00790 (PMC9974066; doi:10.1021/acs.jpclett.2c00790)
Supplement: Supplementary file 1 — jz2c00790_si_001.pdf [file jz2c00790_si_001.pdf]

Supporting Information

Dynamic Instability and Time Domain Response of a Model Halide Perovskite Memristor for Artificial Neurons

Juan Bisquert,*[1] Antonio Guerrero[1]
[1]Institute of Advanced Materials (INAM), Universitat Jaume I, 12006 Castelló, Spain.

Corresponding author J. Bisquert (bisquert@uji.es)

S2

```
Cm0 = 10; Rb = 1; ic0 = 10; τd = 0.1; τk = 100 τd; τm = Rb Cm0;
t0 = 1; Δt = 1; t1 = t0 + Δt; t2 = t1 + 10 Δt; uapp = 1.2; Iapp = Idc[uapp];
{tm, td, tk, u, Ia}
{τm, τd, τk, uapp, Iapp}
{vv1, ic1, fo1}

(* Three dimensional model *)
sol = NDSolve[{
   Cm0 v'[t] == Iapp (HeavisideTheta[t - 1]) - v[t] / Rb - ic[t],
   τd ic'[t] == ic0 fo[t] - ic[t],
   τk fo'[t] == 1 - fo[t] (1 + Exp[- (v[t] - VR) / Vm]),
   v[0] == 0, ic[0] == 0, fo[0] == 0}, {v, ic, fo}, {t, 0, t1}];
vv1 = {Evaluate[v[t1]] /. sol}[[1, 1]]; ic1 = {Evaluate[ic[t1]] /. sol}[[1, 1]];
fo1 = {Evaluate[fo[t1]] /. sol}[[1, 1]];
as1 =
  Show[Plot[{Evaluate[{v[t]} /. sol]}, {t, 0, t1}, PlotRange → {{0, t1}, {-1, 12}},
    PlotStyle → {{AbsoluteThickness[2], Blue}}, BaseStyle -> {FontSize → 16},
    FrameLabel → {Style["t", FontSize → 20], Style["u", FontSize → 20], "", ""},
    Frame → True, LabelStyle → (FontFamily → "Arial")]];

sol = NDSolve[{
    Cm0 v'[t] == -v[t] / Rb - ic[t],
    τd ic'[t] == ic0 fo[t] - ic[t],
    τk fo'[t] == 1 - fo[t] (1 + Exp[- (v[t] - VR) / Vm]),
    v[t1] == vv1, ic[t1] == ic1, fo[t1] == fo1}, {v, ic, fo}, {t, t1, t2}];
as2 = Show[
   Plot[{Evaluate[{v[t]} /. sol]}, {t, t1, t2}, PlotRange → {{t1, t2}, {-8, 12}},
    PlotStyle → {{AbsoluteThickness[2], Blue}}, BaseStyle -> {FontSize → 16},
    FrameLabel → {Style["t", FontSize → 20], Style["u", FontSize → 20], "", ""},
    Frame → True, LabelStyle → (FontFamily → "Arial")]];

jk1 = ParametricPlot[{uv, uapp}, {uv, t0, t1}, PlotRange → {{0, t2}, {0, 3}},
   FrameLabel → {Style["t", FontSize → 20], Style["u", FontSize → 20], "", ""},
   Frame → True, PlotStyle → {{AbsoluteThickness[1], Magenta}},
   LabelStyle → (FontFamily → "Arial"), AspectRatio → 0.7, ImageSize → 90 × 3`];
jk2 = Graphics[{Gray, Line[{{t0, Iapp Rb}, {t0, 0}}]}];
jk3 = Graphics[{Gray, Line[{{t1, Iapp Rb}, {t1, 0}}]}];
jk4 = ParametricPlot[{uv, Iapp Rb}, {uv, t0, t1}, PlotRange → {{t0, t1}, {0, 3}},
   FrameLabel → {Style["u", FontSize → 20], Style["Idc", FontSize → 20], "", ""},
   Frame → True, FrameTicks → {{0, 0.5, 1, 1.5, 2}, {0, 5, 10}, None, None},
   PlotStyle → {{AbsoluteThickness[1], Gray}},
   LabelStyle → (FontFamily → "Arial"), ImageSize → 70 × 2`];

Show[jk1, jk2, jk3, jk4, as1, as2,
 PlotRange → {{0, t2}, {-2, 2}}, BaseStyle -> {FontSize → 16}]
{tm, td, tk, u, Ia}

{10, 0.1, 10., 1.2, 11.0201}
```

{0.998784, 4.14643, 0.0278547}

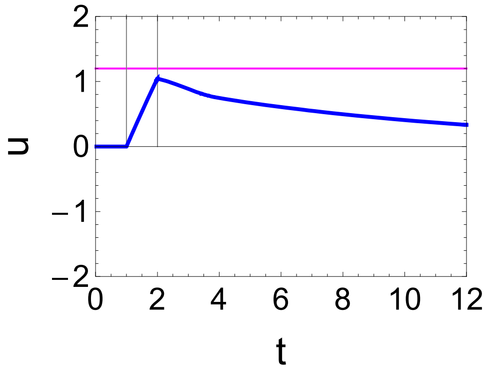

```
(* Two dimensional model*)
Cm0 = 10; Rb = 1; ic0 = 10; τd = 0.1; τk = 100 τd; τm = Rb Cm0;
t0 = 2; Δt = 2; t1 = t0 + Δt; t2 = t1 + 20 Δt;
uapp = 1; Iapp = Idc[uapp];
{tm, td, tk, u, Ia}
{τm, τd, τk, uapp, Iapp}

sol = NDSolve[{
   Cm0 v'[t] == Iapp (HeavisideTheta[t - t0]) - v[t] / Rb - ic[t],
   τd ic'[t] == ic0 / (1 + Exp[- (v[t] - VR) / Vm]) - ic[t],
   v[0] == 0, ic[0] == 0}, {v, ic}, {t, 0, t1}];
vv1 = {Evaluate[v[t1]] /. sol}[[1, 1]]; ic1 = {Evaluate[ic[t1]] /. sol}[[1, 1]];
as1 = Show[
   Plot[{Evaluate[{v[t]} /. sol]}, {t, 0, t1}, PlotRange → {{0, t1}, {-10, 20}},
    PlotStyle → {{AbsoluteThickness[2], Blue}}, BaseStyle -> {FontSize → 16},
    FrameLabel → {Style["t", FontSize → 20], Style["u", FontSize → 20], "", ""},
    Frame → True, LabelStyle → (FontFamily → "Arial")]];

sol = NDSolve[{
    Cm0 v'[t] == -v[t] / Rb - ic[t],
    τd ic'[t] == ic0 / (1 + Exp[- (v[t] - VR) / Vm]) - ic[t],
    v[t0 + Δt] == vv1, ic[t0 + Δt] == ic1}, {v, ic}, {t, t1, t2}];
as2 = Show[
   Plot[{Evaluate[{v[t]} /. sol]}, {t, t1, t2}, PlotRange → {{t1, t2}, {-15, 20}},
    PlotStyle → {{AbsoluteThickness[2], Blue}}, BaseStyle -> {FontSize → 16},
    FrameLabel → {Style["t", FontSize → 20], Style["u", FontSize → 20], "", ""},
    Frame → True, LabelStyle → (FontFamily → "Arial")]];

jk1 = ParametricPlot[{uv, uapp}, {uv, t0, t1}, PlotRange → {{0, t2}, {0, 3}},
   FrameLabel → {Style["t", FontSize → 20], Style["u", FontSize → 20], "", ""},
   Frame → True, PlotStyle → {{AbsoluteThickness[1], Magenta}},
   LabelStyle → (FontFamily → "Arial"), AspectRatio → 0.7, ImageSize → 90 × 3`];
jk2 = Graphics[{Gray, Line[{{t0, Iapp Rb}, {t0, 0}}]}];
jk3 = Graphics[{Gray, Line[{{t1, Iapp Rb}, {t1, 0}}]}];
jk4 = ParametricPlot[{uv, Iapp Rb}, {uv, t0, t1}, PlotRange → {{t0, t1}, {0, 3}},
   FrameLabel → {Style["u", FontSize → 20], Style["Idc", FontSize → 20], "", ""},
   Frame → True, FrameTicks → {{0, 0.5, 1, 1.5, 2}, {0, 5, 10}, None, None},
   PlotStyle → {{AbsoluteThickness[1], Gray}},
   LabelStyle → (FontFamily → "Arial"), ImageSize → 70 × 2`];

Show[jk1, jk2, jk3, jk4, as1, as2,
 PlotRange → {{0, t2}, {0, 2}}, BaseStyle -> {FontSize → 16}]
```

```
{tm, td, tk, u, Ia}

{10, 0.1, 10., 1, 6.}
```

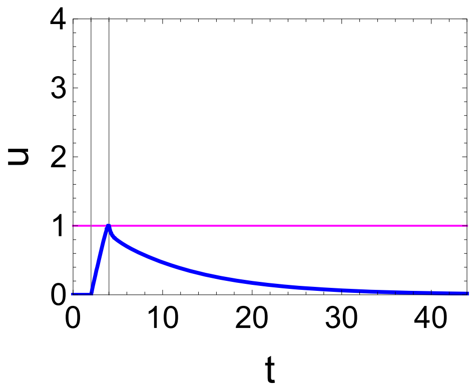


```
Cm = 0.01; Rb = 1; ic0 = 10; τd = 0.01; τk = 10 τd;
VR = 1; Vm = 0.05; AxV1 = 0; AxV2 = 2;  τm = Rb Cm;
ff[u_] := 1 / (1 +  Exp[- (u - VR) / Vm])
Ra[V_] := Vm / ic0 1 / (ff[V] (1 - ff[V]))
La[V_] := ff[V] Ra[V] τk
Idc[u_] := u / Rb + ic0 ff[u]
ZI[up_, w_] := 1 / (1 / Rb + Cm I w + 1 / ((1 + I w τd) (Ra[up] + La[up] I w)))
ZP[up_, w_] := (1 + I w τd) (Ra[up] + La[up] I w)

{tm, td, tk}
{τm, τd, τk}

(*potentiostatic*)
Cm0 = 0.1; Rb = 1; Rs = 0.1; ic0 = 10; τd = 10; τk = 0; τm = Rb Cm0;
t0 = 1; Δt = 3; t1 = t0 + Δt;
Vapp = 1.5; uc = FindRoot[Vapp == Idc[uv] Rs + uv, {uv, 1}][[1, 2]];
Iapp = Idc[uc]
Vapp / Rb
{τm, τd, Δt}

sol = NDSolve[{
   Cm0 v'[t] == Vapp (HeavisideTheta[t - t0]) / Rs - v[t] / Rs - v[t] / Rb - ic[t],
   τd ic'[t] == ic0 / (1 + Exp[- (v[t] - VR) / Vm]) - ic[t],
   v[0] == 0, ic[0] == 0}, {v, ic}, {t, 0, t1}];
vv1 = {Evaluate[v[t1]] /. sol}[[1, 1]]; ic1 = {Evaluate[ic[t1]] /. sol}[[1, 1]];
as1 = Show[Plot[{Evaluate[{(Vapp (HeavisideTheta[t - 1]) - v[t]) / Rs} /. sol]},
    {t, 0, t1}, PlotRange → {{0, t1}, {-10, 30}},
    PlotStyle → {{AbsoluteThickness[2], Blue}}, BaseStyle -> {FontSize → 16},
    FrameLabel → {Style["t", FontSize → 20], Style["I", FontSize → 20], "", ""},
    Frame → True, LabelStyle → (FontFamily → "Arial")]];

sol = NDSolve[{
    Cm0 v'[t] == -v[t] / Rs - v[t] / Rb - ic[t],
    τd ic'[t] == ic0 / (1 + Exp[- (v[t] - VR) / Vm]) - ic[t],
    v[t1] == vv1, ic[t1] == ic1}, {v, ic}, {t, t1, 2 t1}];
as2 = Show[Plot[{Evaluate[{-v[t] / Rs} /. sol]},
    {t, t1, 2 t1}, PlotRange → {{t1, 2 t1}, {-30, 10}},
    PlotStyle → {{AbsoluteThickness[2], Blue}}, BaseStyle -> {FontSize → 16},
    FrameLabel → {Style["t", FontSize → 20], Style["u", FontSize → 20], "", ""},
```

```
   Frame → True, LabelStyle → (FontFamily → "Arial")]];
as3 = Graphics[{AbsoluteThickness[2], Blue,
   Line[{{t1, (Vapp - vv1) / Rs}, {t1, -15}}]}];

jk1 = ParametricPlot[{uv, Iapp}, {uv, t0, t1}, PlotRange → {{0, 2 t1}, {0, 3}},
  FrameLabel → {Style["t", FontSize → 20], Style["I", FontSize → 20], "", ""},
  Frame → True, PlotStyle → {{AbsoluteThickness[1], Magenta}},
  LabelStyle → (FontFamily → "Arial"), AspectRatio → 0.7, ImageSize → 90 × 3`];
jk2 = Graphics[{Gray, Line[{{t0, Vapp / Rs}, {t0, 0}}]}];
jk3 = Graphics[{Gray, Line[{{t1, Vapp / Rs}, {t1, 0}}]}];
jk4 = ParametricPlot[{uv, Vapp / Rs}, {uv, t0, t1}, PlotRange → {{t0, t1}, {0, 3}},
  FrameLabel → {Style["u", FontSize → 20], Style["Idc", FontSize → 20], "", ""},
  Frame → True, FrameTicks → {{0, 0.5, 1, 1.5, 2}, {0, 5, 10}, None, None},
  PlotStyle → {{AbsoluteThickness[1], Gray}},
  LabelStyle → (FontFamily → "Arial"), ImageSize → 90 × 2`];

Show[as1, as2, as3, jk1, jk2, jk3, jk4,
 PlotRange → {{0, 2 t1}, {-12, 20}}, BaseStyle -> {FontSize → 16}]



(*Iu curve*)
tm1 = ParametricPlot[{uv, Log[10, Idc[uv]]}, {uv, -3, 3},
  PlotRange → {{-1, 1}, {-3, 3}}, FrameLabel → {Style["u", FontSize → 20],
    Style["Log I_tot", FontSize → 20], "", ""}, Frame → True,
  FrameTicks → {{-3, -2, -1, 0, 1, 2, 3}, {-2, -1, 0, 1, 2, 3}, None, None},
  PlotStyle → {{AbsoluteThickness[2], Gray}},
  LabelStyle → (FontFamily → "Arial"), AspectRatio → 1, ImageSize → 90 × 4`];
tm2 = ParametricPlot[{uv, Log[10, uv / Rb]}, {uv, -3, 3},
  PlotRange → {{-1, 1}, {-3, 3}}, FrameLabel →
   {Style["u", FontSize → 20], Style["I_tot", FontSize → 20], "", ""}, Frame → True,
  FrameTicks → {{-3, -2, -1, 0, 1, 2, 3}, {-2, -1, 0, 1, 2, 3}, None, None},
  PlotStyle → {{AbsoluteThickness[2], Green}},
  LabelStyle → (FontFamily → "Arial"), AspectRatio → 1, ImageSize → 90 × 4`];
tm3 = ParametricPlot[{uv, Log[10, ic0 ff[uv]]}, {uv, -3, 3},
  PlotRange → {{-1, 1}, {-3, 3}}, FrameLabel → {Style["u", FontSize → 20],
    Style["Log I_tot", FontSize → 20], "", ""}, Frame → True,
  FrameTicks → {{-3, -2, -1, 0, 1, 2, 3}, {-2, -1, 0, 1, 2, 3}, None, None},
  PlotStyle → {{AbsoluteThickness[2], Magenta}},
  LabelStyle → (FontFamily → "Arial"), AspectRatio → 0.8, ImageSize → 90 × 4`];
Show[tm3, tm2, tm1, PlotRange → {{0, 2}, {-1.5, 1.5}},
 BaseStyle -> {FontSize → 16}]


(* Impedance *)
uapp = 0.8;
ty1 = ParametricPlot[{Re[ZI[uapp, 2 π 10^pote]], -Im[ZI[uapp, 2 π 10^pote]]},
  {pote, -4, 4}, PlotRange → {{-1, 1}, {-1, 1}},
  BaseStyle -> {FontSize → 18}, LabelStyle → (FontFamily → "Arial"),
  FrameLabel → {Style["Z'", FontSize → 18], Style["-Z''", FontSize → 18], "", ""},
  Frame → True, FrameTicks → {{-4, -3, -2, -1, -0.5, 0, 0.5, 1, 2, 3, 4, 5},
    {-3, -2, -1, -0.5, 0, 0.5, 1, 2, 3, 4, 5}, None, None}, PlotStyle →
```

```
    {{AbsoluteThickness[2]}}, AspectRatio → Automatic, ImageSize → 100 × 4`];
ty2 = Graphics[{PointSize[0.02], Cyan,
    Point[{Re[ZI[uapp, 0]], -Im[ZI[uapp, 0]]}]}];
Show[ty1, PlotRange → {{-0.1, 1}, {-0.3, 0.6}}]

Manipulate[ParametricPlot[{Re[ZI[up1, 2 π 10^pote]], -Im[ZI[up1, 2 π 10^pote]]},
  {pote, -4, 4}, PlotRange → {{-10^po1, 10^po1}, {-10^po1, 10^po1}},
  LabelStyle → (FontFamily → "Arial"),
  FrameLabel → {Style["Z'", FontSize → 18], Style["-Z''", FontSize → 18], "", ""},
  Frame → True, FrameTicks → {{-1, 0, 1}, {-1, 0, 1}, None, None},
  PlotStyle → {{AbsoluteThickness[2]}}, AspectRatio → 1,
  ImageSize → 100 × 4`], {po1, -2, 2}, {up1, 0, 2}]

(* green 0.005 Blue 0.01 Red 0.05*)

{tm, td, tk}

{0.01, 0.01, 0.1}
```

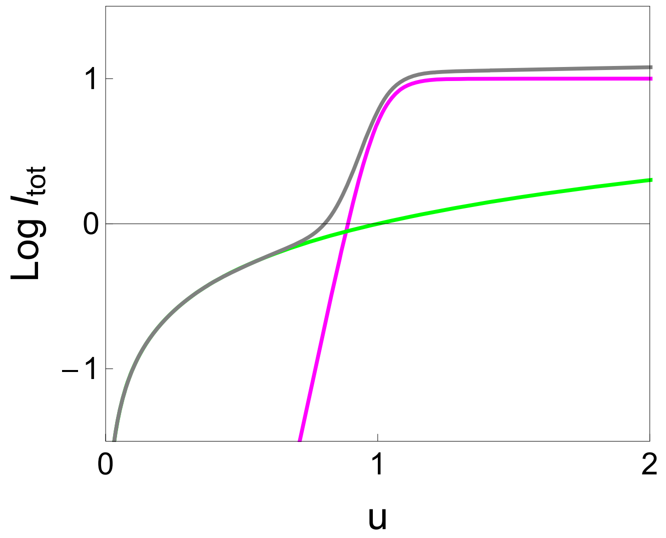

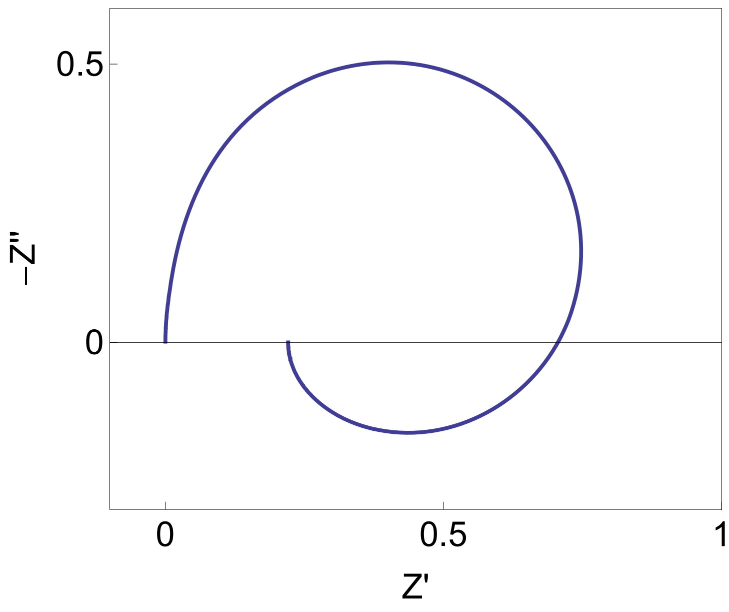
0.5
0
–Z''
0
0.5
1
Z'

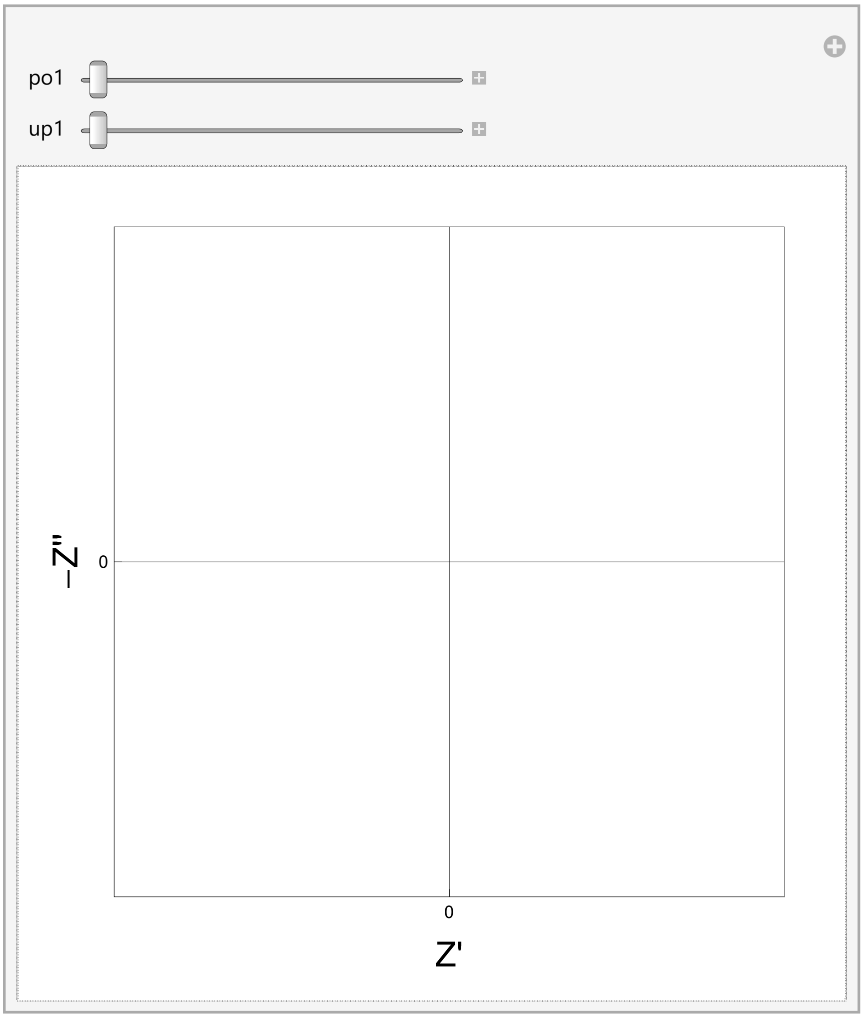
po1
up1
–Z''
0
0
Z'
